# Modeling Alzheimer’s Disease by Induced Pluripotent Stem Cells Carrying *APP* D678H Mutation

**DOI:** 10.1007/s12035-018-1336-x

**Published:** 2018-09-20

**Authors:** Kuo-Hsuan Chang, Guey-Jen Lee-Chen, Ching-Chang Huang, Jia-Li Lin, Yi-Jing Chen, Pei-Chi Wei, Yen-Shi Lo, Chin-Fa Yao, Ming-Wei Kuo, Chiung-Mei Chen

**Affiliations:** 1 Department of Neurology, Chang Gung Memorial Hospital Linkou Medical Center and College of Medicine, Chang Gung University, No.5, Fusing St., Gueishan Township, Taoyuan, 333 Taiwan; 2 Department of Life Science, National Taiwan Normal University, Taipei, Taiwan; 3 Department of Chemistry, National Taiwan Normal University, Taipei, Taiwan; 4 Chang Gung Memorial Hospital Linkou Medical Center, Institute of Stem Cell and Translational Cancer Research, Taoyuan, Taiwan

**Keywords:** Alzheimer’s disease, Induced pluripotent stem cells, Aβ, Tau, *APP*, Neurite outgrowth

## Abstract

Alzheimer’s disease (AD), probably caused by abnormal accumulation of β-amyloid (Aβ) and aberrant phosphorylation of tau, is the most common cause of dementia among older people. Generation of patient-specific neurons by induced pluripotent stem cell (iPSC) technology facilitates exploration of the disease features in live human neurons from AD patients. In this study, we generated iPSCs from two familial AD patients carrying a heterozygous D678H mutation in the *APP* gene (AD-iPSCs). The neurons derived from our AD-iPSCs demonstrated aberrant accumulation of intracellular and secreted Aβ42 and Aβ40, reduction of serine 9 phosphorylation in glycogen synthase kinase 3β (GSK3β) hyperphosphorylation of threonine 181 and serine 396 in tau protein, impaired neurite outgrowth, downregulation of synaptophysin, and increased caspase 1 activity. The comparison between neurons derived from a sibling pair of wild-type and mutated iPSCs successfully recapitulated these AD phenotypes. Treatment with indole compound NC009-1 (3-((1H-Indole-3-yl)methyl)-4-(2-nitrophenyl)but-3-en-2-one), a potential Aβ aggregation reducer, normalized the Aβ levels and GSK3β and tau phosphorylation, attenuated caspase 1 activity, and improved neurite outgrowth in AD-iPSC-derived neurons. Thus, *APP* D678H iPSCs-derived neurons recapitulate the cellular characteristics relevant to AD and enable exploration of the underlying pathogenesis and therapeutic strategies for AD.

## Background

Alzheimer’s disease (AD) is the most prevalent form of dementia associated with progressive cognitive decline and memory loss. The pathological hallmarks of AD include senile plaques and neurofibrillary tangles. Senile plaques are composed of Aβ peptide, a fragment of the amyloid peptide precursor protein (APP) resulting from the cleavage of β- and γ-secretases [1], whereas the main component of neurofibrillary tangles is a cytoskeletal protein known as tau, in a hyperphosphorylated form [2]. Aβ tends to form oligomers and other high-order polymerized structures that may be toxic to neurons [3]. The presence of hyperphosphorylated tau protein also appears to be involved in the neurotoxic process [4]. Currently, the detailed molecular mechanism involved in AD remains to be clarified. Discoveries of fully penetrant mutations in *APP*, *presenilin 1* (*PS1*), and *presenilin 2* (*PS2*) in the autosomal dominant forms of AD have improved our understanding of the potential pathogenesis of AD (review by [5]). Among them, the D678H point mutation in *APP* gene (*APP* D678H, also called Taiwanese mutation) has been identified in autosomal dominant AD families in Taiwan [6, 7].

Currently, any treatment to prevent or slow the rate of AD progression is still lacking. One of the critical limitations for AD research is the paucity of live neurons from patients for a mechanistic approach and drug discovery. Establishment of the human-induced pluripotent stem cells (iPSCs) model has facilitated the generation of patient-specific neurons to study patient-specific characteristics in AD [8]. In this study, we generated iPSCs from two AD patients carrying a heterozygous *APP* D678H mutation (AD-iPSCs). The neurons derived from AD-iPSCs demonstrated aberrant Aβ accumulation and tau phosphorylation, as well as impaired neurite outgrowth. Indole compound NC009-1 (3-((1H-indol-3-yl)methyl)-4-(2-nitrophenyl)but-3-en-2-one) has demonstrated its potential on reduction of Aβ and tau misfolding in 293 and SH-SY5Y AD cell models [9, 10]. Treatment of AD-iPSC-derived neurons with NC009-1 normalized the Aβ levels, tau phosphorylation, and neurite outgrowth. These findings in AD-iPSCs show great promise for modeling human AD in vitro and identifying disease pathogenesis and novel therapeutic targets for AD.

## Materials and Methods

### Derivation of Human Skin Fibroblasts

Fibroblasts were derived from two AD patients (49- year-old female and 54-year-old male) carrying a heterozygous *APP* D678H mutation, as well as a healthy 63-year-old female and 31-year-old male volunteer after they had provided informed consent according to the protocols approved by Chang Gung Memorial Hospital (reference number 102-0597A3). Explants (1 cm^3^) of dermal biopsies were minced with a scalpel into pieces less than 2 mm in diameter. Primary dermal fibroblasts were cultured in medium comprising Glasgow minimum essential medium (GMEM, Invitrogen), 10% fetal calf serum (Invitrogen), 50 U/ml penicillin (Invitrogen), 50 mg/ml streptomycin (Invitrogen), 1 mM sodium pyruvate (Invitrogen), 1× minimum essential medium (MEM) non-essential amino acids (Invitrogen), 2 mM L-glutamine (Invitrogen), and 0.1 mM 2-mercaptoethanol (Invitrogen). Fibroblast cultures were kept at 37 °C in a humidified atmosphere with 5% CO_2_.

### Isolation of Peripheral Blood Mononuclear Cells

For isolation of peripheral blood mononuclear cells (PBMCs), 10~20 ml of peripheral blood was collected by venipuncture donor. Cells were diluted by 2~4× volume of PBS and layered over 20 ml of Ficoll-Hypaque Premium (GE Healthcare) gradient, according to the manufacturer’s instructions, and centrifuged at 1200×*g* for 40 min. The mononuclear cell layer (containing PBMCs) was carefully transferred and washed with PBS for twice. PBMCs wereresuspended in blood medium (1: 1 Iscove's Modified Dulbecco's Medium with glutamine/F12, 5 mg/ml Human serum albumin, 1× lipid concentrate, 10 μg/ml insulin, 100 μg/ml transferrin, 14 ng/ml sodium selenite, 64 μg/ml L-ascorbic acid 2-phosphate, 450 μM 1- thioglycerol, 50 ng/ml SCF, 10 ng/ml IL3, 2 U/ml EPO, 40 ng/ml IGF1, and 1 μM dexamethasone, all bought from Invitrogen) to the density of two to three million cells per ml. Media were changed every 2 or 3 days.

### Preparation of Mouse Embryonic Fibroblasts

Mouse embryonic fibroblasts (MEFs) from 13.5 to 14.5 days post-coitum CD1 mice embryos were generated and cultivated following a standard method to serve as a feeder layer for cultivating human iPSCs [11]. The MEFs were treated with 10 μg/ml mitomycin C (Sigma) for 4 h before use.

### Pluripotent Reprogramming and Culture of Human Induced Pluripotent Stem Cells

Human iPSCs were generated by a retroviral system encoding a combination of four factors including *OCT4* (POU class 5 homeobox 1), *SOX2* (SRY-box 2), *KLF4* (Kruppel-like factor 4), and *c-MYC* (myelocytomatosis oncogene) [12], or by CytoTune™-Sendai viral vector kit (Life Technologies). The human iPSCs were maintained on mitomycin C-treated MEF feeder cells in Knockout Dulbecco’s modified Eagle medium (DMEM, Invitrogen) supplemented with 20% Knockout serum replacement (KSR, Invitrogen), 8 ng/ml bFGF (Peprotech), 50 U/ml penicillin, 50 mg/ml streptomycin, 1 mM sodium pyruvate, 1× MEM non-essential amino acids, 2 mM L-glutamine, and 0.1 mM 2-mercaptoethanol and passaged every week with 0.1% EDTA (Invitrogen) in PBS at a ratio of 1:3.

### Monolayer Neuronal Differentiation

The iPSC colonies were disaggregated using Accutase (Millipore) for 20 min and pre-plated on gelatin for 1 h at 37 °C to remove the MEFs. The non-adhered iPSCs were then plated on Matrigel (BD)-coated plates in MEF-conditioned medium, spiked with 8 ng/ml of bFGF at a density of 10,000–25,000 cells/cm^2^. The iPSCs were expanded in the cell medium for 3 days or until they were nearly confluent. Nearly confluent cells were differentiated in N2B27 medium (1:1 mixture of DMEM/F12 supplemented with modified N2 and neurobasal medium supplemented with B27, all purchased from Invitrogen) supplemented with 10 μM TGF-β inhibitor SB431542 (Sigma) for 7 days. Neural stem cells were generated by transferring the differentiated cells into N2B27 media supplemented with 10 ng/ml bFGF and 10 ng/ml EGF (Peprotech) on poly-L-lysine (Sigma)-coated plates. To generate mature neurons, EGF and bFGF were removed from the N2B27 media on day 12, and the remaining differentiation was completed in maturation medium supplemented with 20 ng/ml BDNF (Peprotech), 0.2 mM ascorbic acid (Sigma), 10–20 ng/ml GDNF (Peprotech), 1 ng/ml TGFb3 (Peprotech), and 0.5–1.0 mM dibutyryl cAMP (Sigma) from days 12 to 42. The media were changed every 2–3 days. Neurons were treated with 5 μM NC009-1 for 7 days.

### Karyotyping Number of Chromosomes

Cells in a confluent T25 flask were treated with 1 μg/ml colchicine (Sigma) for 2.5 h at 37 °C and then dissociated with 0.025% trypsin. The cell suspension was centrifuged at 1300 rpm for 3 min. After washing with PBS, the cells were resuspended in 3 ml PBS and 7 ml 37.5 mM KCl hypotonic solution for 20 min at 37 °C, and then centrifuged at 1300 rpm for 3 min. The pellet was resuspended in 5 ml 3.75 mM KCl solution. After another centrifugation at 1300 rpm for 3 min, the pellet was gently resuspended in 5 ml methanol/acetic acid (3:1, *v*/*v*) and incubated for 30 min at room temperature. The cell suspension was then centrifuged at 1300 rpm for 3 min, and the pellet was resuspended in 200 μl methanol/acetic acid. Two to three drops of cell suspension were dropped onto a microscope slide, and the slide was then air-dried and mounted with Vectershield containing DAPI (Vector Laboratories). The chromosome spread was then photographed under a Leica DMRB epifluorescence microscope.

### Teratoma Formation and Histology

Nude mice were anesthetized with diethyl ether. iPSCs were suspended in PBS containing 1% fetal calf serum at 1 × 10^7^ cells/ml, and 100 μl of the cell suspension was injected subcutaneously into the dorsal flank of mice. Four weeks after the injection, tumors were surgically dissected from the mice, fixed in 4% formaldehyde, and embedded in paraffin. Sections were stained with hematoxylin and eosin.

### Antibodies

The primary antibodies used for immunocytochemistry in this study were rabbit anti-Aβ42 (1:35, Millipore), mouse anti-NES (nestin) (1:500, R&D), rabbit anti-OCT4 (1:500, Abcam), mouse anti-SYP (synaptophysin) (1:500, Abcam), mouse anti-TRA-1-81 (1:250, BD Pharmingen), and rabbit anti-TUBB3 (tubulin β3 class III) (1:1000, Covance). The secondary antibodies included Alexa594-conjugated donkey anti-rabbit IgG (1:200, Invitrogen), Alexa488-conjugated donkey anti-goat IgG (1:200, Invitrogen), and Alexa488-conjugated donkey anti-mouse IgG (1:200, Invitrogen). The antibodies used for western blotting were mouse anti-GSK3β (glycogen synthase kinase 3β) (1:500, Cell Signaling Technology), mouse anti-phospho-GSK3β (Ser9) (1:500, Cell Signaling Technology), mouse anti-tau (1:500, Abcam), mouse anti-phospho-tau (Ser396) (1:1000, sigma), mouse anti-phospho-tau (Thr181) (1:500, Cell Signaling Technology), and mouse anti-TUBB3 (1:10000, Biolegend). The secondary antibodies used included goat anti-mouse IgG-HRP (horseradish peroxidase) (1:20000, GE Healthcare), goat anti-rabbit IgG-HRP (1:20000, GE Healthcare), and donkey anti-goat IgG-HRP (1:2500, Santa Cruz). The immune complexes were detected using an enhanced chemiluminescent substrate (ECL, Biotools).

### *APP* Genotyping and Sequencing

Genomic DNA was isolated from peripheral leukocytes using a DNA Extraction Kit (Stratagene). The *APP* genotype was determined by PCR amplification, gel purification, *Hin*fI digestion, and direct sequencing using an ABI PRISM 3730 Genetic Analyzer (Applied Biosystems). Each PCR included 20 ng of genomic DNA, 0.9 μM of each primer (Table 1), and Universal PCR Master Mix (Applied Biosystems). The PCR conditions were as follows: 95 °C for 5 min, followed by 40 cycles of 95 °C for 40 s, 58 °C for 30 s, and 72 °C for 40 s, with a final extension at 72 °C for 10 min.

**Table 1:** Sequences of primers used in this study

Genes	Forward	Reverse
*β-ACTIN (qRT-PCR)*	ACTCTTCCAGCCTTCCTTCC	GTTGGCGTACAGGTCTTTGC
*APP*	GAGTGCACATGGAAAAAGACAC	GGATTACCATGAAAACATGAAGAA
Exo-*KLF4*	AGGCACACCTGCGAACCCAC	TGGACTAATCCGGATCTCTCG
Endo-*KLF4*	CTTACTCGCCTTGCTGATTG	GCCGAGATCCTTCTTCTTTG
Total-*KLF4*	TGAGCAGCAGGGACTGTCAC	TAATGGAGCGGCGGGACTTG
Exo-*MYC*	CTTGAACAGCTACGGAACTC	TGGACTAATCCGGATCTCTCG
Endo-*MYC*	ATTCCAGCGAGAGGCAGAGG	TTTCGTGGATGCGGCAAGGG
Total-*MYC*	GTGACCAGATCCCGGAGTTG	CTGCTTGGACGGACAGGATG
Exo-*OCT4*	GGCTCTCCCATGCATTCAAAC	TGGACTAATCCGGATCTCTCG
Endo-*OCT4*	TTCGCAAGCCCTCATTTCAC	CGAGAAGGCGAAATCCGAAG
Total-*OCT4*	GGAGGAAGCTGACAACAATG	TCTCACTCGGTTCTCGATAC
Exo-*SOX2*	ATGTCCCAGCACTACCAGAG	TGGACTAATCCGGATCTCTCG
Endo-*SOX2*	GTTGTCAAGGCAGAGAAGAG	GAGGCAGCAAACTACTTTCC
Total-*SOX2*	TCGCCCACCTACAGCATGTC	CGAACCCATGGAGCCAAGAG

### Reverse Transcription Polymerase Chain Reaction

One microgram of RNA was reverse transcribed into complementary DNA (cDNA) using a SuperScript III™ Super Mix kit (Invitrogen) according to the manufacturer’s instructions. RT-PCR was performed using the KAPA SYBR® FAST qPCR Master Mix (Kapa Biosystems). Briefly, 2 μl of 1:10 diluted cDNA was mixed with 10 μl KAPA SYBR® FAST qPCR Master Mix, 10 μM forward primer, 10 μM reverse primer, and 6 μl water. Amplification was performed for 40 cycles (94 °C for 1 min, 65 °C for 1 min, and 72 °C for 1 min). The primer sequences used in this study are shown in the supporting information of Table 1.

### Alkaline Phosphatase Assay

Colonies showing alkaline phosphatase activity were detected using an alkaline phosphatase detection kit (Millipore) according to the manufacturer’s instructions.

### Trypan Blue Exclusion Assay

Cell suspensions (20 μl) were mixed with 20 μl 0.4% trypan blue (Gibco) for 1 h. Cell viability was determined by light microscopy. Cells that excluded trypan blue were considered viable.

### Caspase 1 Activity Assay

Caspase 1 activity was determined using a Caspase Activity Assay Kit (Abcam) according to the manufacturer’s instructions.

### Caspase 3 Activity Assay

Caspase 3 activity was determined using a Caspase Activity Assay Kit (Sigma) according to the manufacturer’s instructions.

### Quantification of Aβ

Cells (1 × 10^5^) were seeded on poly-L-lysine-coated plates and cultured in 1 ml N2B27 medium for 3 days. Cell lysates and medium were collected to determine the intracellular and secreted levels of Aβ40 and Aβ42 using the Aβ40 and Aβ42 Human ELISA Kit (Invitrogen).

### β- and γ-Secretase Activity Assay

β- and γ-secretase activities were determined using β- and γ-secretase activity kits (Abcam) according to the manufacturer’s instructions.

### Neurite Outgrowth Measurement

Neurite outgrowth features of differentiated neurons including total outgrowth, processes, and branches were assessed by MetaMorph microscopy automation and image analysis software (molecular devices) based on the immunocytochemistry of the neuronal marker TUBB3.

### Data Analysis

Experiments for quantification were performed in triplicate. Neurite outgrowth measurements were obtained by counting at least 200 neurons in each independent experiment. Each set of values was expressed as means ± standard deviation. Differences between groups were determined using a two-sample Student’s *t* test or one-way ANOVA with Bonferroni’s *post hoc* test. All *p* values were two-tailed, and a *p* value less than 0.05 was considered to be statistically significant. All statistical analyses were performed using the Statistical Program for Social Science software version 23.

## Results

### Establishment of Induced Pluripotent Stem Cells from Familial AD Patients Carrying the *APP* D678H Mutation and Differentiation of iPSCs to Neurons

**Fig. 1:**
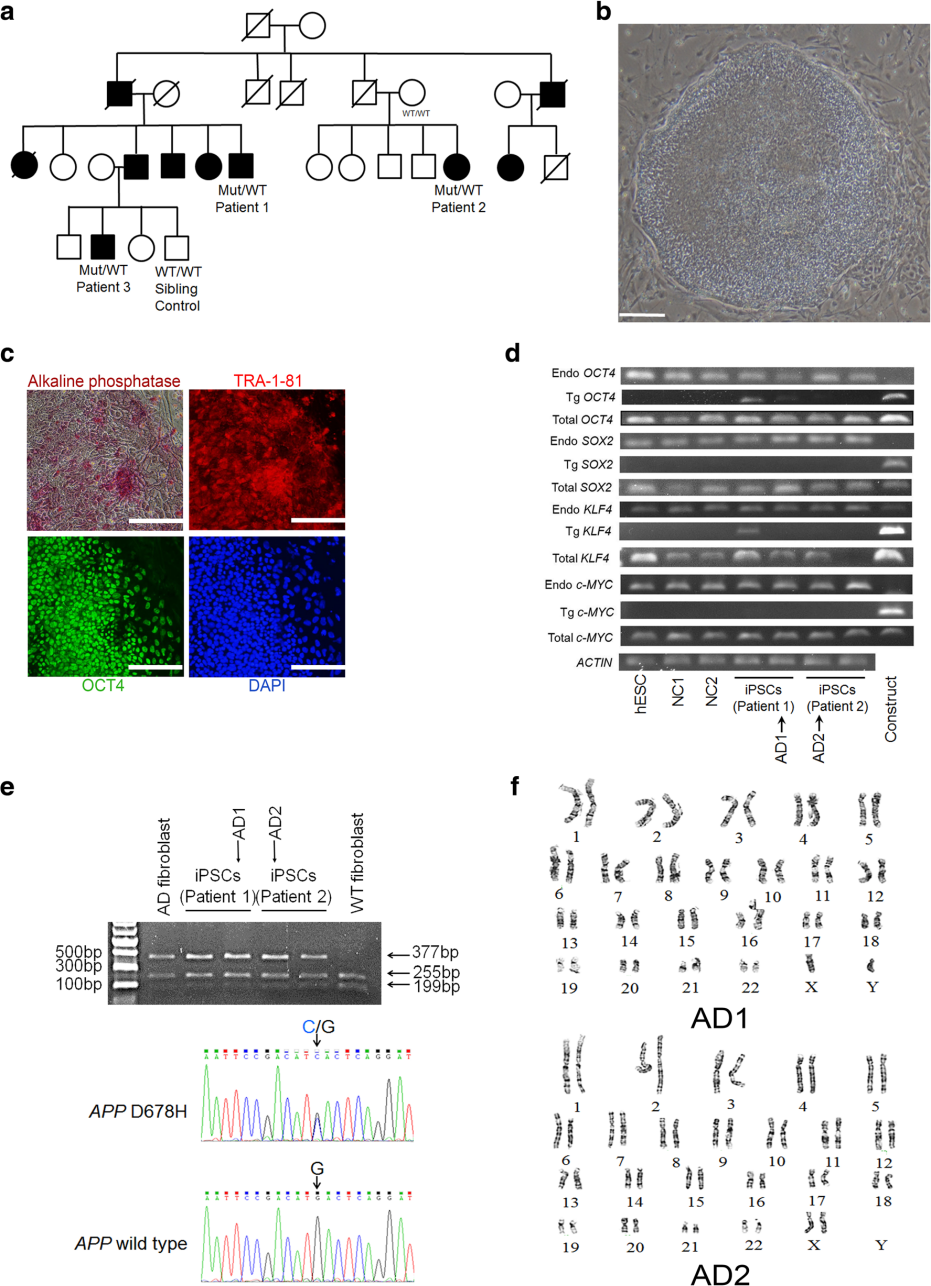
Generation of induced pluripotent stem cells from Alzheimer’s patients carrying *APP* D678H mutation (AD-iPSCs). **a** Pedigree diagram of family with *APP* D678H. Individuals affected with Alzheimer’s disease (AD) and unaffected individuals are represented with black symbols and open symbols, respectively. AD-iPSCs were derived from patients 1–3. WT wild-type, Mut mutation (*APP* D678H). **b** Bright field image and **c** alkaline phosphatase/immunofluorescence staining for OCT4 (green) and TRA-1-81 (red) in AD-iPSCs. Nuclei were stained with DAPI (blue). Scale bar, 100 μm. **d** Expression levels of integrated (Tg-), endogenous (Endo-), and total (Total-) reprogramming gene factors (*OCT4*, *SOX2*, *KLF4*, and *MYC*) in iPSCs derived from a healthy control (NC1~NC2), AD-iPSCs (AD1~AD2), and human embryonic stem cells (hESCs). Construct was used as a negative control for endogenous expression and a positive control for integrated expression. **e** The D678H mutation in *APP* from the AD-iPSCs by genotyping and sequencing. **f** Karyotype analysis of AD-iPSCs

Two patients (patient 1 54-year-old male, patient 2 49-year-old female, see pedigree in Fig. 1a) with early-onset (at 20 years of age) familial AD carrying the *APP* D678H mutation and two healthy volunteers (control 1: 31-year-old male, control 2: 63-year-old female) assented to skin biopsies for derivation of iPSCs. After expanding the primary dermal fibroblast lines, the cells were transduced with retroviruses carrying four reprogramming factors, *OCT4*, *SOX2*, *KLF4*, and *MYC* [12]. Approximately 2 months post-infection, four iPSC clones from the patients with AD (AD-iPSCs) (two clones for each patient) and four clones from the healthy volunteers (NC-iPSCs) (two clones for each control) were established under standard human embryonic stem cell (hESC) culture conditions. These iPSCs formed dense hESC-like colonies (Fig. 1b), expressed the pluripotent hESC marker alkaline phosphatase, were immunoreactive for pluripotent markers OCT4 and TRA-1-81 (Fig. 1c), and were successfully cultured in an undifferentiated state for more than 35 passages (10 months). Two AD-iPSC (AD1: from patient 1, AD2: from patient 2) and two NC-iPSC clones (NC1: from control 1, NC2: from control 2) that lacked expression of the four transgenes were selected for further analysis (Fig. 1d). RT-PCR performed on total RNA and cDNA sequencing confirmed the heterozygous *APP* D678H mutation in the AD-iPSCs (Fig. 1e). All these iPSC clones demonstrated normal chromosome karyotypes (Fig. 1f).

**Fig. 2:**
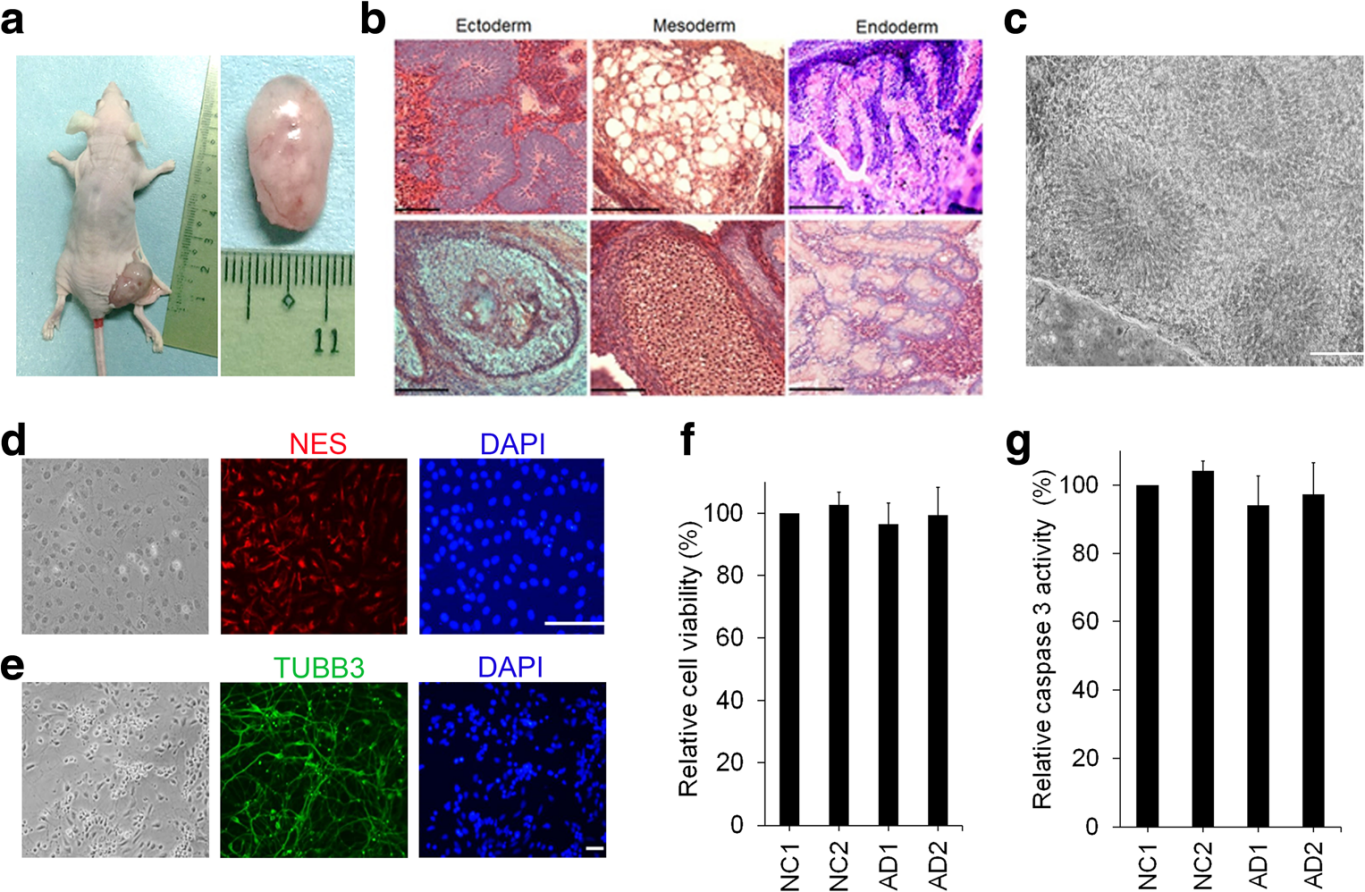
Characterization and neuronal differentiation of AD-iPSCs. **a** Teratoma formed after subcutaneously injecting AD-iPSCs into nude mice. **b** Ectodermal (neural tissues and hair follicles), mesodermal (adipose tissues and cartilages), and endodermal (respiratory and gastrointestinal epithelium) tissues in the teratoma. Scale bar, 100 μm. **c** AD-iPSCs were differentiated into neural rosettes. Scale bar, 100 μm. **d** AD-iPSC-derived neural stem cells expressing NES (red). **e** AD-iPSC-derived neurons expressing TUBB3 (green). Nuclei were stained with DAPI. Scale bar, 100 μm. **f** Relative cell viability and caspase 3 activity of NC- (NC1 and NC2) and AD-iPSC-derived neurons (AD1 and AD2)

These iPSC clones also formed subcutaneous teratomas in nude mice (Fig. 2a, b). Using a feeder-free, chemically defined in vitro neural differentiation protocol, we differentiated the four clones of iPSCs to neurons. These cells formed colonies with an elongated columnar neural rosette morphology following 2 weeks of differentiation (Fig. 2c). They further generated neural stem cells expressing NES (Fig. 2d), and neurons expressing TUBB3 were generated following 6 weeks of differentiation (Fig. 2e). Cell viability and caspase 3 activity of the AD- and NC-iPSC-derived neurons were similar (Fig. 2f, g).

### Accumulation of Aβ in Alzheimer’s Disease-Induced Pluripotent Stem Cell-Derived Neurons

**Fig. 3:**
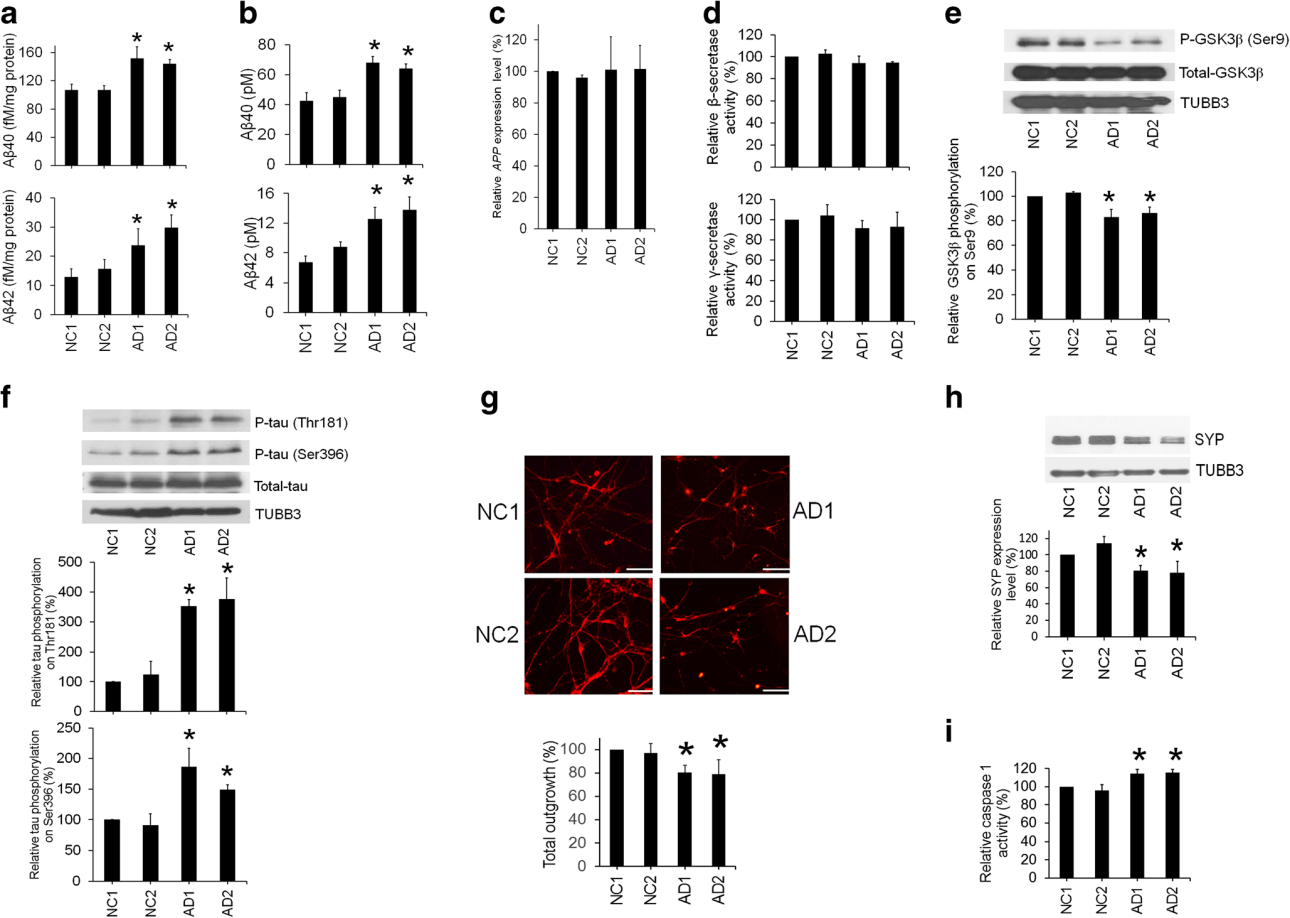
Increased Aβ accumulation and tau phosphorylation, as well as impaired neurite outgrowth in AD-iPSC-derived neurons. **a** Increased levels of intracellular and **b** secreted Aβ40 and Aβ42 in AD-iPSC-derived neurons (AD1 and AD2) compared to NC-iPSC-derived neurons (NC1 and NC2). **c** Expression levels of *APP* in NC- and AD-iPSC-derived neurons. **d** β- and γ-secretase activities in NC- and AD-iPSC-derived neurons. **e** Lower levels of GSK3β phosphorylation (P-GSK3β, serine 9), and **f** higher levels of tau phosphorylation (P-tau, threonine 181, and serine 396) in AD-iPSC-derived neurons compared to NC-iPSC-derived neurons. **g** Reduction of neurite outgrowth. **h** Lower SYP expression levels. **i** Higher caspase 1 activities in AD-iPSC-derived neurons compared to NC-iPSC-derived neurons. Data were normalized to NC1. *p < 0.05 compared to NC1 and NC2

Intracellular and extracellular amyloidogenic Aβ depositions are major pathological findings in the brains of AD patients [13]. To determine whether AD-iPSC-derived neurons recapitulated these critical phenotypes of AD, we examined the Aβ levels in the AD-iPSC-derived neurons and found higher levels of Aβ40 and Aβ42 in neurons derived from AD-iPSCs than in those from NC-iPSCs (Fig. 3a). However, we did not observe any intracellular aggregate formation. We also examined the levels of secreted Aβ and found that AD-iPSCs released higher concentrations of Aβ40 and Aβ42 compared to NC-iPSCs (Fig. 3b). qRT-PCR showed that AD- and NC-iPSC-derived neurons had similar transcriptional levels of *APP* (Fig. 3c). The activities of β- and γ-secretase in AD- and NC-iPSC-derived neurons were identical (Fig. 3d). These results excluded the potential contribution of *APP* transcriptional or processing deregulation and suggested the enhancement of amyloidogenic cleavage of *APP* caused by the *APP* D678H mutation.

### Reduction of GSK3β Phosphorylation and Increase of Tau Phosphorylation in Alzheimer’s Disease-Induced Pluripotent Stem Cell-Derived Neurons

The presence of Aβ increases GSK3β activity [14], which subsequently hyperphosphorylates tau protein [15]. We found that serine 9 phosphorylation in GSK3β, which inhibits GSK3β activity [16], was significantly reduced in AD-iPSC-derived neurons (Fig. 3e). The phosphorylation sites threonine 181 and serine 396 in tau protein were also hyperphosphorylated in AD-iPSC-derived neurons compared with NC-iPSC-derived neurons (Fig. 3f). These results indicate the downstream signaling alterations resulted from Aβ accumulation in AD-iPSC-derived neurons.

### Impairment of Neurite Outgrowth in Alzheimer’s Disease-Induced Pluripotent Stem Cell-Derived Neurons

Hyperphosphorylation of tau can be crucial for preventing the interaction of tau protein with microtubules and affects microtubule stabilization and dynamics that are essential to neurite outgrowth [15]. Therefore, we compared neurite outgrowth levels between neurons derived from AD- and NC-iPSCs. AD-iPSC-derived neurons demonstrated impaired neurite outgrowth compared to NC-iPSC-derived neurons (Fig. 3g). The expression levels of the neurite marker SYP were also downregulated in AD-iPSC-derived neurons compared to NC-iPSC-derived neurons (Fig. 3h). Caspase 1 is known to be highly expressed in the AD brain and is associated with axonal degeneration [17]. Our results showed that AD-iPSC-derived neurons expressed higher activities of caspase 1 (Fig. 3i) compared to NC-iPSC-derived neurons, successfully capturing this important phenotype of axonal degeneration in AD.

### Recapitulation of Alzheimer’s Phenotypes by Comparing a Sibling Pair of Induced Pluripotent Stem Cell-Derived Neurons

**Fig. 4:**
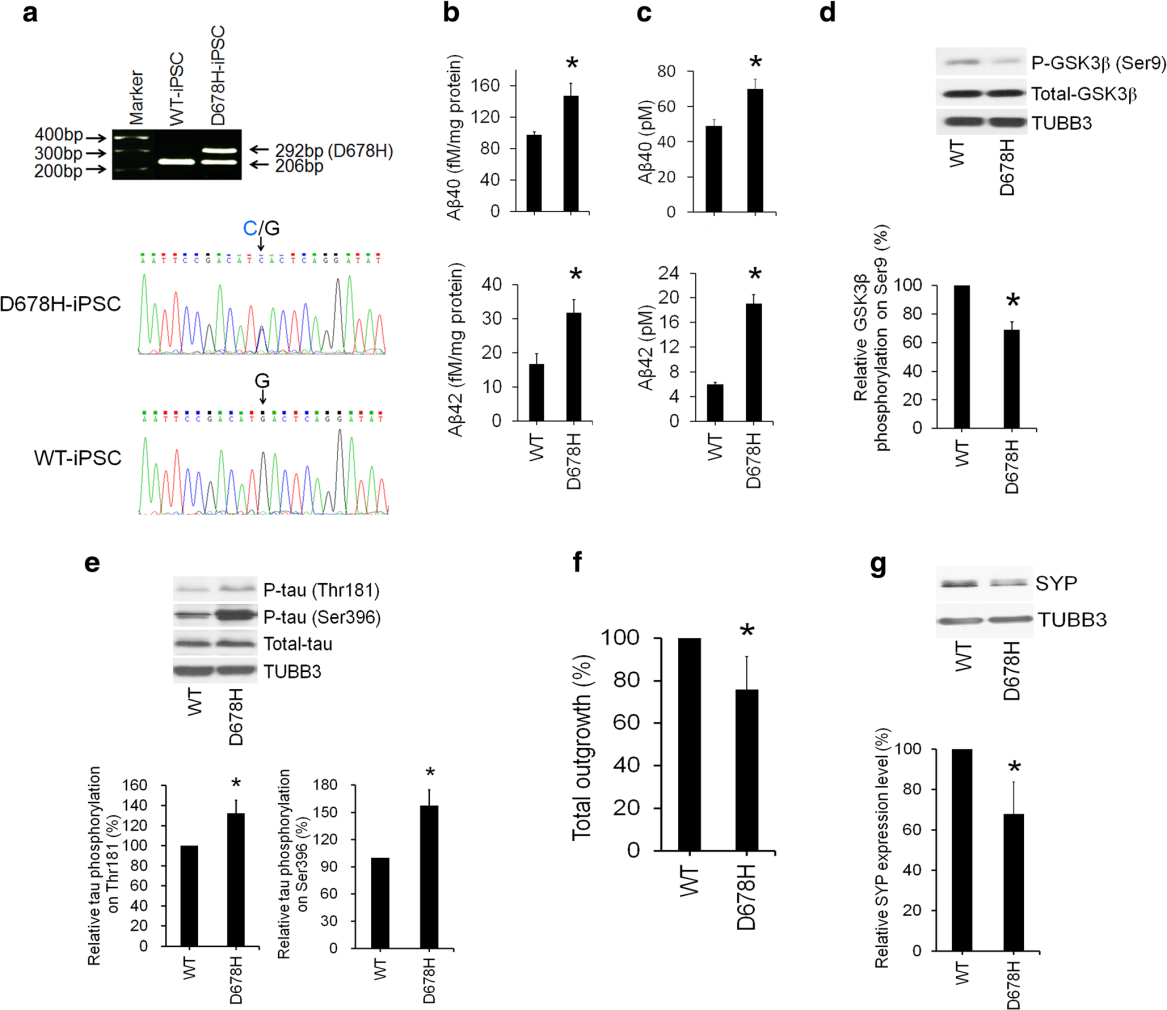
Comparison of iPSC-derived neurons from a sibling pair of a wild-type control subject and an *APP* D678H-mutated patient. **a** Genotyping of a sibling pair of wild-type and *APP* D678-mutated iPSCs. **b** Increased levels of intracellular and **c** Aβ40 and Aβ42. **d** Lower levels of GSK3β phosphorylation (serine 9). **e** Higher levels of tau phosphorylation (threonine 181 and serine 396). **f** Reduction of neurite outgrowth. **g** Lower expression levels of SYP in neurons derived from iPSCs carrying *APP* D678H mutation (D678H) compared to those derived from wild type-iPSCs (WT). **c** Increased levels of secreted Aβ40 and Aβ42 in neurons derived from iPSCs carrying *APP* D678H mutation compared to those derived from wild type-iPSCs. Data were normalized to WT. *p < 0.05 compared to WT-iPSC-derived neurons

To further confirm the above phenotypes, we generated iPSCs from PBMCs of a male patient carrying *APP* D678H mutation (D678H-iPSC, derived from patient 3 in Fig. 1a) and his brother (WT-iPSC, derived from sibling control in Fig. 1a) by Sendai virus reprogramming method (Fig. 4a). Similar to previous results, the levels of intracellular and secreted Aβ40/Aβ42 were higher in the neurons derived from iPSCs carrying *APP* D678H compared to those derived from WT-iPSCs (Fig. 4b, c). Neurons derived from *APP* D678H-mutated iPSCs also present with lower level of serine 9 phosphorylation in GSK3β (Fig. 4d), as well as higher threonine 181 and serine 396 in tau protein compared to WT-iPSC-derived neurons (Fig. 4e). Neurite outgrowth analysis displayed worse total outgrowth in neurons derived from *APP* D678H-mutated iPSCs compared to WT-iPSC-derived neurons (Fig. 4f). The expression levels of SYP were lower in neurons derived from *APP* D678H-mutated iPSCs compared to WT-iPSC-derived neurons (Fig. 4g). These results successfully recapitulated and validated the neuronal phenotypes in our AD-iPSC model.

### Improvement of Alzheimer’s Disease Phenotypes by Indole Compound NC009-1 in the Alzheimer’s Disease-Induced Pluripotent Stem Cell Model

**Fig. 5:**
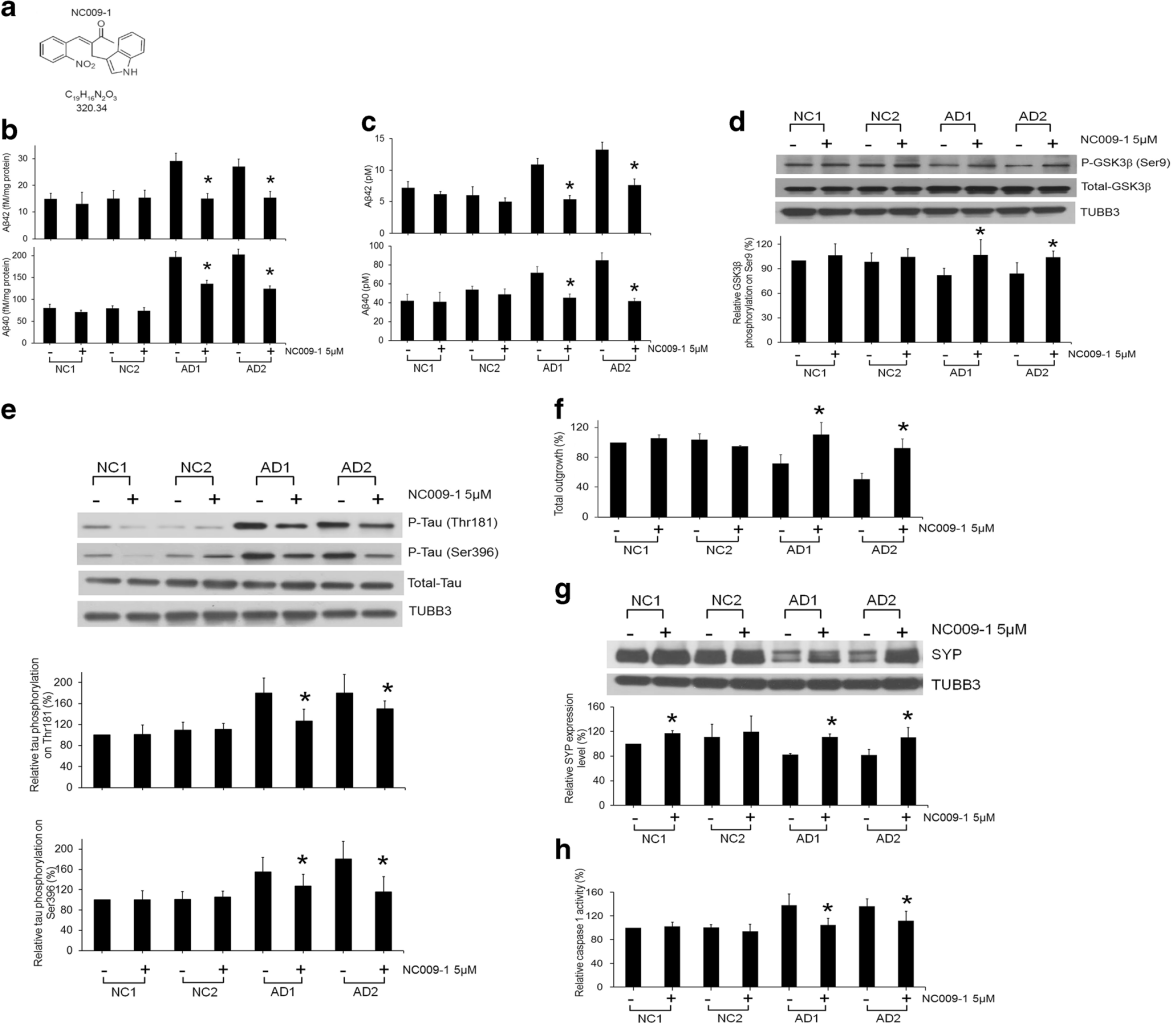
Treatment with indole compound NC009-1 improved AD phenotypes in AD-iPSC-derived neurons. **a** Structure, formula, and molecular weight of indole compound NC009-1. **b** Intracellular and **c** secreted levels of Aβ40 and Aβ42, **d** GSK3β (serine 9), and **e** tau (threonine 181 and serine 396) phosphorylation levels. **f** Neurite outgrowth. **g** SYP expression levels. **h** Caspase 1 activities in AD- (AD1 and AD2) and NC-iPSC-derived neurons (NC1 and NC2) upon treatment with NC009-1. Data were normalized to NC1. *p < 0.05 compared to neurons without NC009–1 treatment

Previously, we found that synthetic indole compound NC009-1 (Fig. 5a) improved neuronal cell viability, neurite outgrowth, and SYP expression levels in mouse hippocampal primary cultures under oligomeric Aβ-induced neurotoxicity [9]. To evaluate its therapeutic potential in AD patient-specific neurons, we treated AD- or NC-iPSC-derived neurons with 5 μM NC009-1 for 7 days. Treatment with NC009-1 efficiently decreased the intracellular and secreted levels of Aβ40/Aβ42 levels in AD-iPSC-derived neurons (Fig. 5b, c). The reduction of serine-9 phosphorylation in AD-iPSC-derived neurons was significantly improved by treatment with NC009-1 (Fig. 5d). Furthermore, NC009-1 reduced the hyperphosphorylation of threonine-181 and serine 396 in tau protein in AD-iPSC-derived neurons (Fig. 5e). The impairment of total outgrowth, SYP downregulation, and high caspase 1 activities were improved by treatment with NC009-1 (Fig. 5f–h). These findings indicate the potential of NC009-1 in rescuing the AD phenotypes in our AD-iPSC model.

## Discussion

In this study, we established an AD-iPSC model that recapitulated the crucial phenotypes of AD from two familial AD patients carrying the *APP* D678H mutation. In addition to aberrant accumulation of Aβ and tau phosphorylation, this AD-iPSC model demonstrated important features of impaired neurite outgrowth accompanied with SYP downregulation and high caspase 1 activity. Treatment with indole compound NC009-1 significantly reduced Aβ accumulation and tau phosphorylation, improved neurite outgrowth and the expression level of SYP, and decreased the activity of caspase 1. Our AD-iPSCs not only provide a model of AD to explore disease phenotypes at the cellular level but also serve as a platform for the development of disease-modifying agents for AD in the future.

*APP* mutations account for a minority of familial AD patients. Mutations near the cleavage site of α-secretase (E693K, E693G, E693del) enhance the proteolytic resistance of Aβ [18]. *APP* mutations at codon 714–715, near the γ-secretase cleavage site, decrease the secretion of Aβ40, and the mutations at codon 716–717 increase the production and secretion of Aβ42 [18]. Abnormal Aβ accumulation has also been reported in iPSC-derived neurons from AD patients carrying *APP* V717I mutation [19], duplicated *APP* [20], as well as those with Down syndrome [21]. We did not find any differences in the expression levels of *APP* in our AD-iPSC-derived neurons, excluding the possibility that *APP* expression was altered by long-standing differentiation of the cells, or by the *APP* D678H mutation. We further examined the activities of β- and γ-secretase, which were normal in AD-iPSC-derived neurons. Since the *APP* D678H mutation lies near the β-secretase cleavage site of APP, this mutation may cause an APP conformation change, which may enhance the amyloidogenic cleavage of APP and lead to Aβ accumulation in AD-iPSCs. On the other hand, the nearby *APP* D678N and H677R mutations may accelerate the formation of Aβ fibrils without affecting Aβ production [7, 22, 23]. Therefore, different *APP* mutations may generate AD phenotypes through different mechanisms. Our AD-iPSC model provides insights into the pathogenic mechanism of AD caused by the *APP* D678H mutation.

Aβ accumulation prevents phosphorylation-mediated GSK3β deactivation [24]. GSK3β has been proposed as the link between Aβ aggregation and tau pathology as it modifies tau phosphorylation [15]. Abnormal tau phosphorylation and activation of GSK3β has been revealed in iPSC-derived neurons from familial AD patients carrying duplicated *APP* and a limited number of sporadic AD patients [20]. IPSC-derived neurons from patients with Down syndrome also show abnormal tau phosphorylation [21, 25]. Here, we recapitulate the abnormal tau phosphorylation and GSK3β activity in iPSC-derived neurons carrying the *APP* D678H mutation. These findings, for the first time, demonstrate cellular tauopathy in AD-iPSC models carrying a point mutation in the *APP* gene, and also provide a platform to identify therapeutic compounds targeting tauopathy.

Tau is a neuronal microtubule-associated protein that facilitates microtubule assembly in vitro [26], thus playing an important role in microtubule dynamics [27] and also in axonal transport [28]. In addition to altered APP processing, an increase in levels of total and phosphorylated tau has been observed in iPSC-derived neurons carrying *APP* V717I mutation [19]. GSK3β activation increased tau phosphorylation and neurite retraction [29, 30]. Thus, impairment of neurite outgrowth in AD-iPSC-derived neurons carrying *APP* D678H in our study represents this neurite defect. However, we did not find significant neuronal death in our AD-iPSC model. Given that axonopathy is known as an early event in AD [31], it is possible that our AD-iPSC-derived neurons model the pathological features in the early stage of AD. Therefore, other pathological hallmarks such as Aβ aggregation, neurofibrillary tangles, and neuronal apoptosis that appear in the later stage of disease are still absent in this AD-iPSC model. Stimulating factors, such as progerin overexpression or oxidative stress [32, 33], may accelerate the aging process to capture these pathological features in the advanced stage of AD patients. However, given that therapeutics administered to AD patients in early stage are more likely to have significant beneficial effects, our AD-iPSCs may serve as a more useful model than those capturing the late pathological features.

Indole compounds are present in a wide variety of biologically active compounds including Aβ aggregation inhibitors [34]. By reducing Aβ and tau misfolding, indole compound NC009-1 has demonstrated its potential to improve neurite outgrowth and neuronal viability in in vitro and ex vivo models of AD [9, 10]. Here, we further demonstrated its potential to normalize Aβ accumulation, GSK3β activity, tau phosphorylation, and caspase 1 activity, as well as improve neurite outgrowth in iPSC-derived neurons carrying the *APP* D678H mutation. These findings support the potential of indoles in treating AD, thus reinforcing the application of AD-iPSC models as a platform for pre-clinical drug discovery in AD.

Considerable inter- and intra-sample variations have been noticed and should be minimized by sibling or isogenic comparisons in the research of iPSC models. Take advantage of patients with constitutional mosaicism for Down syndrome, isogenic comparison of neurons derived from trisomy 21 and euploid iPSC lines showed increased production of Aβ in Down syndrome iPSC-derived neurons [35]. Here, we generated a sibling pair of control and mutant iPSC lines. The sibling comparison of neurons derived from WT- and *APP* D678H-mutated iPSCs consolidates the generations of AD phenotypes including aberrant Aβ accumulation and tau phosphorylation, as well as impaired neurite outgrowth by *APP* D678 mutation. Such phenotypes are more likely to be robust, reproducible, and therefore more amenable to developing into high-throughput screening assays for biomarker and drug research.

Neurons derived from iPSCs carrying *APP*-D678H mutations reveal key features that may be useful in mechanistic studies and small-molecule screening in AD research. However, the heterogeneous nature of AD may limit the generalization of these neuroprotective strategies to all AD patients. Our * in vitro* neuronal differentiation may only mimic AD phenotypes at the pre-clinical or early stage of disease. Important pathological hallmarks in AD patients at advanced stages, such as Aβ aggregation and neurofibrillary tangles, are still absent in our model. The interaction between different cell types may not be explored in our monolayer neuronal culture system but can be achieved using iPSC-derived brain organoids in the future. Nevertheless, our study provides an important disease model to identify potential disease-modifying strategies individually and potentially elucidates the future development of personalized medicine for patients with AD.

## Abbreviations

Aβ: β-amyloid
AD: Alzheimer’s disease
APP: Amyloid peptide precursor protein
DMEM: Dulbecco’s modified Eagle medium
GMEM: Glasgow minimum essential medium
GSK3β: Glycogen synthase kinase 3β
iPSC: Induced pluripotent stem cell
KLF4: Kruppel-like factor 4
KSR: Knockout serum replacement
MEF: Mouse embryonic fibroblast
MEM: Minimum essential medium
c-MYC: Myelocytomatosis oncogene
NES: nestin
OCT4: POU class 5 homeobox 1
PS1: Presenilin 1
PS2: Presenilin 2
RT-PCR: Reverse transcription polymerase chain reaction
SOX2: SRY-box 2
SYP: synaptophysin
TUBB3: Tubulin β3 class III
